# Endoplasmic reticulum—shape and function in stress translation

**DOI:** 10.3389/fpls.2014.00425

**Published:** 2014-09-01

**Authors:** Federica Brandizzi, Lorenzo Frigerio, Stephen H. Howell, Patrick Schäfer

**Affiliations:** ^1^Plant Research Laboratory, Department of Energy, Michigan State UniversityEast Lansing, MI, USA; ^2^School of Life Sciences, University of WarwickCoventry, UK; ^3^Department of Genetics, Development and Cell Biology, Plant Sciences Institute, Iowa State UniversityAmes, IA, USA

**Keywords:** unfolded protein response (UPR), ER associated degradation (ERAD), ER stress, bZIP transcription factors, myosins, caspases, ER bodies, cysteine endopeptidase, programmed cell death

The endoplasmic reticulum (ER) is a very versatile organelle. Besides its major role as the gateway to the secretory pathway the ER is central to adaptation against abiotic and biotic stress. Here, we summarize the current knowledge on ER dynamics and architecture, the association and interaction of the ER with other organelles as well as its role in stress translation and adaptation (Figure 1).

**Figure 1:**
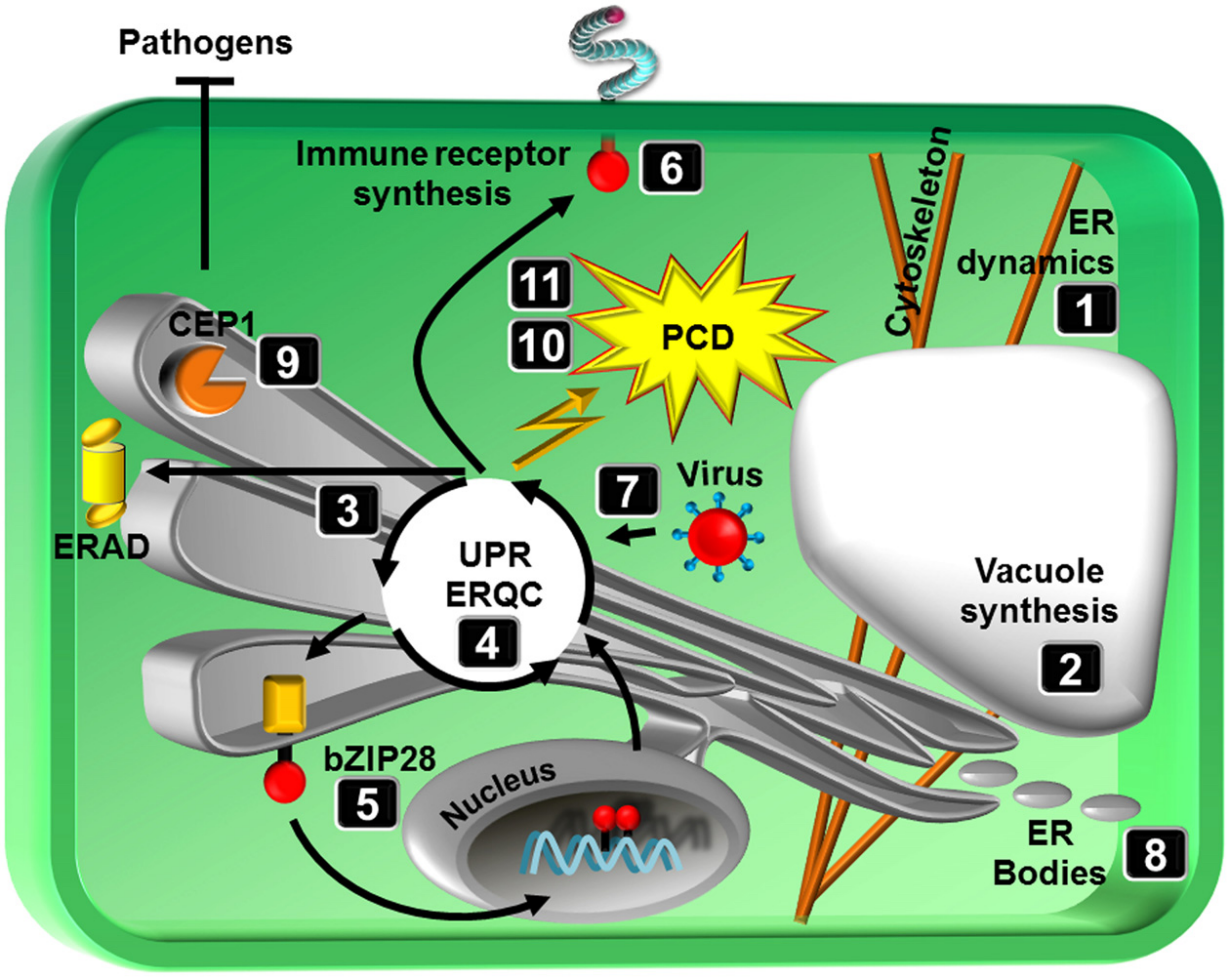
**Schematic representation of topics covered in the special issue**. Numbers correspond to review articles as follows: (1) Griffing et al. (2014), ER network dynamics are differentially controlled by myosins XI-K, XI-C, XI-E, XI-I, XI-1, and XI-2, (2) Viotti (2014), ER and vacuoles: never been closer, (3) Li and Liu (2014), Endoplasmic reticulum-mediated protein quality control in Arabidopsis, (4) Ruberti and Brandizzi (2014), Conserved and plant-unique strategies for overcoming endoplasmic reticulum stress, (5) Srivastava et al. (2014), Stress sensing in plants by an ER stress sensor/transducer, bZIP28, (6) Tintor and Saijo (2014), ER-mediated control for abundance, quality, and signaling of transmembrane immune receptors in plants, (7) Verchot (2014), The ER quality control and ER associated degradation machineries are vital for viral pathogenesis, (8) Nakano et al. (2014), ER bodies in plants of the Brassicales order: biogenesis and association with innate immunity, (9) Höwing et al. (2014), Endoplasmic reticulum KDEL-tailed cysteine endopeptidase 1 of Arabidopsis (AtCEP1) is involved in pathogen defense, (10) Williams et al. (2014), When supply does not meet demand-ER stress and plant programmed cell death, (11) Cai et al. (2014), Endoplasmic reticulum stress-induced PCD and caspase-like activities involved.

The ER is a dynamic network of membrane tubules and sheets. Movement of the ER along the actin cytoskeleton has a significant influence on both its architecture and dynamics. Members of class XI myosin motor proteins have recently been shown to control the movement of the ER and other organelles such as Golgi bodies, peroxisomes and mitochondria. Griffing et al. (2014) explore a subset of class XI myosins with a focus on their role in regulating ER network remodeling by affecting ER tubulation, sheet formation and the persistence of these structures. In addition to the importance of these ER network dynamics for organelle communication, the ER may further directly provide the source membrane for vacuole biogenesis, as hypothesized by Viotti (2014). The majority of soluble vacuolar proteins travel from the ER to the Golgi complex. A growing number of reports however indicate that some proteins, including some vacuolar membrane proteins appear to reach the vacuole without visiting intermediate compartments of the endomembrane system.

An essential function of the ER is the synthesis of secreted proteins. To insure that only correctly folded proteins are exported from the ER, eukaryotes have evolved ER quality control (ERQC) mechanisms, which supervise the folding process. Protein folding is monitored through the stepwise modification of oligosaccharide side chains on glycoproteins, and proteins that fail to fold correctly are extracted from the folding process and subjected to ER associated degradation (ERAD). Li and Liu (2014) describe the protein folding process and how client proteins are chosen for ERAD. Proper function of the ER is particular relevant under stress where the demand for secreted proteins exceed the ER working load capacity. Under such conditions, ER homeostasis requires cellular communication between the ER and the nucleus. Therefore, eukaryotes have signaling proteins on the ER membrane that sense impaired ER function (ER stress) through the accumulation of misfolded proteins in the ER and that employ different strategies to signal the nucleus. Some of these communication strategies are common to all eukaryotes, while others are unique to plants. Ruberti and Brandizzi (2014) compare the ER stress signal pathways between yeast, plants and animals and review the responses in plants, which vary from adaptive measures to cell death. The bZIP transcription factor bZIP28 represents an ER stress signaling factor that is tethered to the ER membrane under unstressed conditions but is mobilized and transferred to the nucleus upon stress. Srivastava et al. (2014) describe the structure of bZIP28 and underlying principles of bZIP28 mobilization in response to stress.

ER stress activates the unfolded proteins response (UPR) and the ERAD system to eliminate misfolded proteins, which is vital for the establishment of an effective immune system. Tintor and Saijo (2014) introduce the significance of the ERQC in the synthesis of immune receptors and provide further insights into the causalities of ER function (UPR) and plant immunity. Despite its role in immunity, the ER has been hijacked by viruses to promote viral pathogenesis. As reviewed by Verchot (2014), viruses have developed sophisticated strategies to overload the ER protein folding machinery with viral encoded proteins in infected cells. Interestingly, this activates the UPR and up-regulates cellular chaperones that further aid in virus protein folding. The overall significance of the ER in stress adaption is however apparent and underlined by the review of Nakano et al. (2014) which, in addition to the biogenesis and evolution, discusses the putative function of ER bodies in abiotic and biotic stress. As ER-derived compartments ER bodies contain stress-associated proteins (e.g., various β-glucosidases of the PYK10 family) that are thought to enhance stress resilience by activating glucosinolates and glucosylated phytohormones. The study of Höwing et al. (2014) further identified an immune-active function of the ER-localized cysteine endopeptidase AtCEP1. In addition to its function in developmental processes, AtCEP1 is expressed and localized to the ER network, which condenses around haustoria during powdery mildew infection. Knockout of AtCEP1 results in enhanced susceptibility, suggesting that AtCEP1 is involved in restricting fungal infection, possibly by controlling a defense-related programmed cell death (PCD) at the late stages of the interaction.

Cells mount adaptive responses (e.g., UPR) to mitigate the damage caused by ER stress. However, when stress becomes so excessive that the ERQC system cannot meet the demands, then PCD ensues. Williams et al. (2014) review the possible links between the UPR and PCD and discuss the involvement of calcium signaling and N-rich proteins in promoting PCD and the role of ER chaperones in limiting it as well as the connection of pathogens to ER-mediated cell death. Cai et al. (2014) finally compare the activation and/or regulation of ER stress-induced PCD in animals and plants and highlight the significance of caspase and caspase-like activities in underlying PCD processes.

The articles published in this e-book summarize our current knowledge of the multi-functionality of the ER in stress adaptation. By providing a stable microenvironment for the synthesis of metabolites and secreted proteins, the ER functions as an intracellular stress sensory organelle and, accordingly, initiates and regulates adaptive responses to environmental stress. Understanding the molecular basis of these processes and the role of ER architectural dynamics therein is of high relevance for sustainable crop production. We hope our e-book will stimulate research in the field to further enhance our knowledge of ER biology in plants.

## Conflict of interest statement

The authors declare that the research was conducted in the absence of any commercial or financial relationships that could be construed as a potential conflict of interest.

## References

[B1] Cai Y. M. Yu J. Gallois P. (2014). Endoplasmic reticulum stress-induced PCD and caspase-like activities involved. Front. Plant Sci. 5:41 10.3389/fpls.2014.00041 24592269 PMC3924713

[B2] Griffing L. Gao H. T. Sparkes I. (2014). ER network dynamics are differentially controlled by myosins XI-K, XI-C, XI-E, XI-I, XI-1, and XI-2. Front. Plant Sci. 5:218 10.3389/fpls.2014.00218 24904614 PMC4033215

[B3] Höwing T. Huesmann C. Hoefle C. Nagel M. K. Isono E. Hückelhoven R. (2014). Endoplasmic reticulum KDEL-tailed cysteine endopeptidase 1 of Arabidopsis (AtCEP1) is involved in pathogen defense. Front. Plant Sci. 5:58 10.3389/fpls.2014.00058 24605116 PMC3932416

[B4] Li J. Liu Y. (2014). Endoplasmic reticulum-mediated protein quality control in Arabidopsis. Front. Plant Sci. 5:162 10.3389/fpls.2014.00162 24817869 PMC4012192

[B5] Nakano R. T. Yamada K. Bednarek P. Nishimura M. Hara-Nishimura I. (2014). ER bodies in plants of the Brassicales order: biogenesis and association with innate immunity. Front. Plant Sci. 5:73 10.3389/fpls.2014.00073 24653729 PMC3947992

[B6] Ruberti C. Brandizzi F. (2014). Conserved and plant-unique strategies for overcoming endoplasmic reticulum stress. Front. Plant Sci. 5:69 10.3389/fpls.2014.00069 24616733 PMC3935401

[B7] Srivastava R. Deng Y. Howell S. H. (2014). Stress sensing in plants by an ER stress sensor/transducer, bZIP28. Front. Plant Sci. 5:59 10.3389/fpls.2014.00059 24616727 PMC3935173

[B8] Tintor N. Saijo Y. (2014). ER-mediated control for abundance, quality, and signaling of transmembrane immune receptors in plants. Front. Plant Sci. 5:65 10.3389/fpls.2014.00065 24616730 PMC3933923

[B9] Verchot J. (2014). The ER quality control and ER associated degradation machineries are vital for viral pathogenesis. Front. Plant Sci. 5:66 10.3389/fpls.2014.00066 24653727 PMC3949406

[B10] Viotti C. (2014). ER and vacuoles: never been closer. Front. Plant Sci. 5:20 10.3389/fpls.2014.00020 24550928 PMC3913007

[B11] Williams B. Verchot J. Dickman M. (2014). When supply does not meet demand-ER stress and plant programmed cell death. Front. Plant Sci. 5:211 10.3389/fpls.2014.00211 24926295 PMC4045240

